# The diagnostic value of lymphocyte profiling for infectious mononucleosis and mycoplasma pneumoniae pneumonia

**DOI:** 10.3389/fped.2025.1607857

**Published:** 2025-08-01

**Authors:** Xuan Shen, Wenya Li, Jijin Qi, Qing Zhong, Yaoyun Zhang, Tianyu Sun, Siqi Shi, Liang Zhang, Jing Su

**Affiliations:** ^1^ Department of Hematology, Jiangsu Province (Suqian) Hospital, Suqian, Jiangsu, China; ^2^ Department of Nephrology, Jiangsu Province (Suqian) Hospital, Suqian, Jiangsu, China

**Keywords:** lymphocyte subsets, infectious mononucleosis (IM), mycoplasma pneumoniae pneumonia (MPP), CD4/CD8, ROC

## Abstract

**Background:**

Infectious mononucleosis (IM) and mycoplasma pneumoniae pneumonia (MPP) are common pediatric diseases that can easily trigger immune responses. This study aims to detect peripheral blood lymphocyte subsets through flow cytometry and explore the diagnostic value in IM and MPP.

**Methods:**

This retrospective study using electronic medical records included a total of 86 children with IM, 150 children with MPP, and 120 healthy volunteers. Lymphocyte subsets in peripheral blood were quantitatively detected by flow cytometry, covering lymphocyte ratio, CD8+, CD4+, CD3+, CD4/CD8, CD19, and CD16 + cells. The diagnostic value of these markers was analyzed using receiver operating characteristic (ROC) curve.

**Results:**

Compared with the HC and IM groups, the MPP group had a significantly lower lymphocyte ratio (*P* < 0.01). In the MPP group, CD8 + and CD3 + lymphocyte proportions were significantly reduced (*P* < 0.01), while CD4+/CD8 + ratio, CD4 + and CD19+ B cell levels were significantly higher than those of the IM and HC groups (*P* < 0.01). In the comparison between MPP and IM, ROC analysis showed that CD3+ T cells (AUC = 0.931), CD3 + CD8+ T cells (AUC = 0.989), CD4/CD8 ratio (AUC = 0.996), CD3 + CD4+ T cells (AUC = 0.992), lymphocyte ratio (AUC = 0.925), and CD19+ B cells (AUC = 0.978) all had excellent diagnostic value.

**Conclusion:**

Peripheral blood lymphocyte subset analysis could serve as a precise and efficient diagnostic tool to directly distinguish between MPP and IM.

## Introduction

Infectious mononucleosis (IM) is a disease caused by Epstein–Barr virus (EBV). The primary mode of infection is via saliva transmission. Infection with EBV is common in children, and the initial infection typically presents as IM, especially more prevalent during adolescence (1, 2). It is currently widely accepted that a significant increase in the number of EBV-specific CD8 + cells is a notable feature of IM. Variations in the proportions of other cell subsets has not been fully clarified (3). In IM, peripheral blood lymphocyte subsets exhibit dynamic alterations during disease progression (4). EBV preferentially targets B cells, inducing their necrosis or apoptosis. Concurrently, CD8+ T cells are activated to eliminate virus-infected cells (5–9), thereby contributing to a reduction in the CD4+/CD8 + ratio (4, 10). While CD4 + and CD8+ T cells are critical in preventing viral reactivation from latency, the precise mechanisms underlying this regulatory function remain incompletely understood and warrant further investigation (11). Although the acute symptoms of IM are typically self-limiting and can usually resolve on their own without specific antiviral therapy, serious complications may occur without appropriate clinical monitoring and supportive care (12).

Mycoplasma pneumoniae pneumonia (MPP) is a significant cause of community-acquired pneumonia in children, comprising 10%–40% of cases, with most occurrences in children over five years old (13, 14). Mycoplasma pneumonia is a significant pathogen responsible for human pneumonia. It has a high incidence rate and is frequently observed in children experiencing respiratory infections. It frequently occurs in children with respiratory tract infections, having a high incidence rate. It can severely affect children's growth, development, and overall health (15). This disease is mostly of the subacute type. In the early stage, the main symptoms are headache and an irritating dry cough. multi-organ dysfunction and death can occur as the disease progresses (16). Neutrophils play a key role in innate immunity and are closely related to the development of inflammatory responses (17). Past research has found that in children with sMPP, the number of neutrophils in peripheral blood increases, and there is also a significant infiltration of neutrophils in lung tissue. The worsening of MPP is associated with immune reactions mediated by neutrophils (18–20). When the neutrophil count is lower it usually corresponds to a milder degree of disease (21).

The pathophysiology of IM and MPP in children is closely related to the immune response, and lymphocyte subsets play a key role in this process. Recent meta-analyses have further highlighted the potential diagnostic and prognostic utility of flow cytometry-based immune profiling in pediatric infections (22, 23). Although both IM and MPP involve significant immune activation, the underlying mechanisms differ: a constitutive attribute of IM is a robust CD8+ T-cell response driven by EBV-infected B cells, whereas the pathogenesis of MPP involves neutrophil-mediated inflammation and an unbalanced CD4 + -dominated T-cell response. There is a lack of simple clinical indicators to assess the prognosis of IM and MPP in children. There is an urgent clinical need to find simple and effective clinical indicators to accurately distinguish between IM and MPP in children.

This study used flow cytometry to detect peripheral blood lymphocyte subsets in children with IM and MPP. It aimed to explore the diagnostic value of different lymphocyte subsets for IM and MPP patients to distinguish between these two diseases.

## Materials and methods

### General information

**Figure 1:**
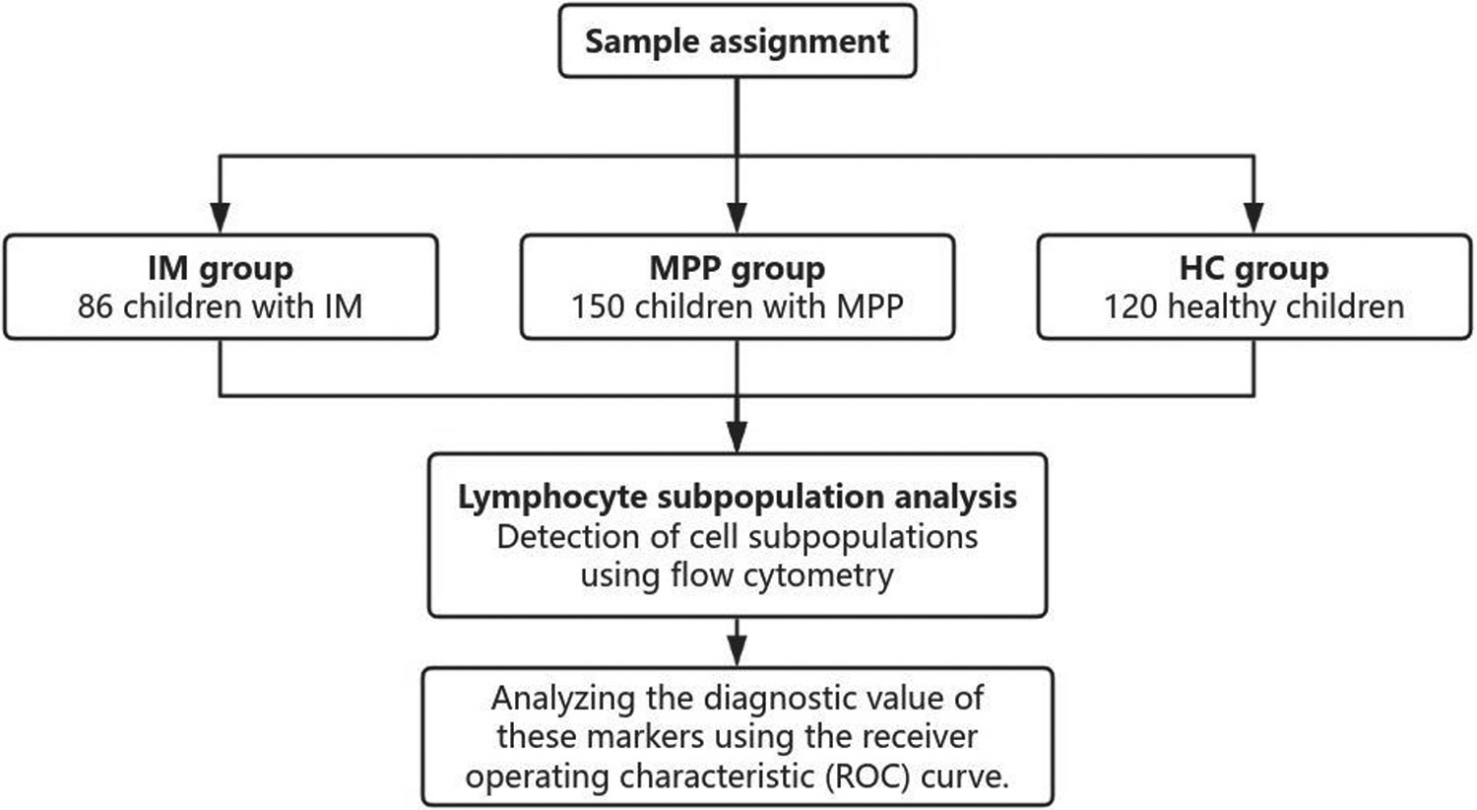
Flow chat.

The retrospective study collected the electronic medical records of a total of 356 patients between December 2022 and December 2024 including 86 patients with IM, 150 patients with MPP and 120 health controls (HC) (Figure 1). Healthy controls were children undergoing routine health check-ups at Suqian Hospital during the same study period with no active infections or immunological disorders. The study was approved by the Medical Ethics Committee of Suqian Hospital (2025-SR-0058), and informed consent was obtained from all patients’ parents or legal guardians.

### Inclusion criteria and exclusion criteria

#### Inclusion criteria

Inclusion criteria for IM required the presence of any three of the following clinical indicators: (1) eyelid edema; (2) pharyngotonsillitis; (3) splenomegaly; (4) cervical lymph node enlargement; (5) fever; (6) hepatomegaly. In addition, patients with IM must fulfill at least one of the following criteria: (1) more than fourfold increase in serum EBV VCA IgG from baseline; (2) positive blood EBV DNA PCR; and (3) positive serum EBV VCA IgM antibody (24).

Inclusion criteria for MPP were as follows: (1) all children included in the study had not received any prior antimycoplasma pneumoniae or anti-infection treatments. (2) the study subjects exhibited symptoms such as cough, fever, and sputum expectoration. (3) diagnosis of MPP was confirmed by a combination of clinical criteria, including chest imaging (such as CT scans) showing unilateral pneumonia and positive results from Mycoplasma pneumoniae-specific nucleic acid tests conducted on nasopharyngeal aspirates (18, 25).

#### Exclusion criteria

The exclusion criteria are as follows: (1) Patients with hematological malignancies or terminal solid tumors; (2) Absence of complete blood count data within 24 hours of admission; (3) Patients who have received organ transplantation, or used hormones, cytotoxic agents, or immunosuppressive drugs within the past two weeks; (4) Patients who choose to stop treatment, discharge themselves, or transfer to other hospitals; (5) Hospitalization duration of less than 24 hours.

### Research methodology

#### Reagents and instruments

The fluorescent antibodies including CD3-FITC, CD16 + CD56-PE, CD45-PerCP-Cy5.5, CD4-PC7, CD19-APC, and CD8APC-Cy7 were acquired from Beijing Tongsheng Times Biotechnology Co. The samples were analyzed on a NAVIOS dual-laser, eight-color flow cytometer (Beckman, USA).

#### Flow cytometry analysis

2 ml of fasting venous blood was collected within 8 h of patient admission and heparin sodium was added to prevent clotting. Take 100 μl of the anticoagulated blood sample and use the microsphere counting tube and add the following reagents (20 μl each) respectively: CD3, CD16 + CD56, CD45, CD4, CD19 and CD8. Allow the mixture to incubate for 15 minutes at room temperature. Add 2.0 ml of lysing agent to the mixture and incubate in the dark at room temperature for an additional 10 minutes. After incubation, centrifuge the mixture at 300 g for 5 minutes and discard the supernatant. Wash the cells twice with phosphate-buffered saline (PBS), each time adding 1 ml of PBS. Resuspend the processed cell sample and transfer it to a flow cytometer, following the instrument's operating instructions. Analyze 15,000 lymphocytes using flow cytometry to evaluate their fluorescent signals and use the data to accurately determine the percentages of lymphocyte subpopulations in peripheral blood. Data was collected and analyzed using Navios software and the statistical software.

### Statistical analysis

All statistical analyses were performed using SPSS 27.0. Homogeneity of variance testing showed statistically significant variance differences (*P* < 0.05). Tamhane's test was used for non-homogeneous groups, and LSD analysis for homogeneous groups (*P* > 0.05). Data are presented as mean ± SD. One-way ANOVA was used for multiple group comparisons, with *P* < 0.05 indicating statistical significance. Receiver operating characteristic (ROC) curve analysis was performed using SPSS 27.0 to assess the diagnostic potential of peripheral blood immune cell ratios. The *p*-values in ROC analysis denote the statistical significance of the AUC against a null hypothesis value of 0.5 (AUC > 0.8 indicates diagnostic utility).

## Result

### Comparison of lymphocyte composition in the IM, MPP and HC groups

A total of 86 children with IM, 150 children with MPP, and 120 healthy control (HC) children were included in this study (Table 1). Compared with the HC and IM groups, the MPP group exhibited a significantly lower lymphocyte ratio (*P* < 0.01) (Table 1). In the MPP group, the proportions of CD8 + and CD3 + lymphocytes were significantly reduced (*P* < 0.01) (Table 1). The levels of CD4 + and CD19+ B cells in the MPP group were significantly elevated (*P* < 0.001) (Table 1). The CD4+/CD8 + ratio of the MPP group was significantly higher than those of the IM and HC groups (*P* < 0.01) (Table 1). There were no significant differences in the proportion of CD3-CD16 + CD56 + between the MPP group and the IM group, as well as between the MPP group and the HC group (*P* > 0.05) (Table 1).

**Table 1:** Comparison of lymphocyte subsets and clinical characteristics among MPP, IM and HC groups

Variable	MPP (*n* = 150)	IM (*n* = 86)	HC (*n* = 120)	*P* (MPPvsIM)	*P* (MPPvsHC)
Age, years	4.53 ± 3.02	4.74 ± 2.35	2.29 ± 1.03	0.339	<0.001
Gender, *n* (%)				0.826	0.891
Male	85 (56.7%)	50 (58.1%)	69 (57.5%)		
Female	65 (43.3%)	36 (41.9%)	51 (42.5%)		
Immunological markers					
CD3 + (%)	66.7 (60.7,72.7)	84.1 (79.6,86.7)	75.1 (69.5,85.1)	<0.001	<0.001
CD4 + (%)	35.9 (29.0,40.0)	13.6 (10.1,18.2)	21.3 (13.3,37.1)	<0.001	<0.001
CD8 + (%)	24.0 (18.1,28.8)	61.6 (52.8,69.0)	38.4 (21.5,64.0)	<0.001	<0.001
CD4/CD8	1.5 (1.1,2.2)	0.21 (0.15,0.34)	0.51 (0.19,1.77)	<0.001	<0.001
CD3-CD16 + CD56 + (%)	9.9 (6.9,14.5)	8.7 (7.0,11.5)	8.7 (7.1,11.9)	0.486	0.450
CD19 + (%)	19.3 (14.4,23.5)	4.3 (3.0,6.6)	10.2 (4.1,17.4)	<0.001	<0.001
Lymphocyte ratio (%)	31.5 (22.4,41.4)	63.9 (56.9,68.9)	52.9 (46.4,59.2)	<0.001	<0.001
Inflammatory parameters					
WBC	8.5 (6.7,11.5)	15.3 (11.4,20.6)	7.2 (6.3,8.9)	<0.001	<0.001
CRP	8.4 (4.5,13.7)	7.4 (3.0,12.7)	4.3 (2.4,7.3)	0.155	0.155
PCT	0.07 (0.05,0.15)	0.17 (0.10,0.36)	0.05 (0.02,0.42)	<0.001	0.015

### The diagnostic value of lymphocyte subsets

**Figure 2:**
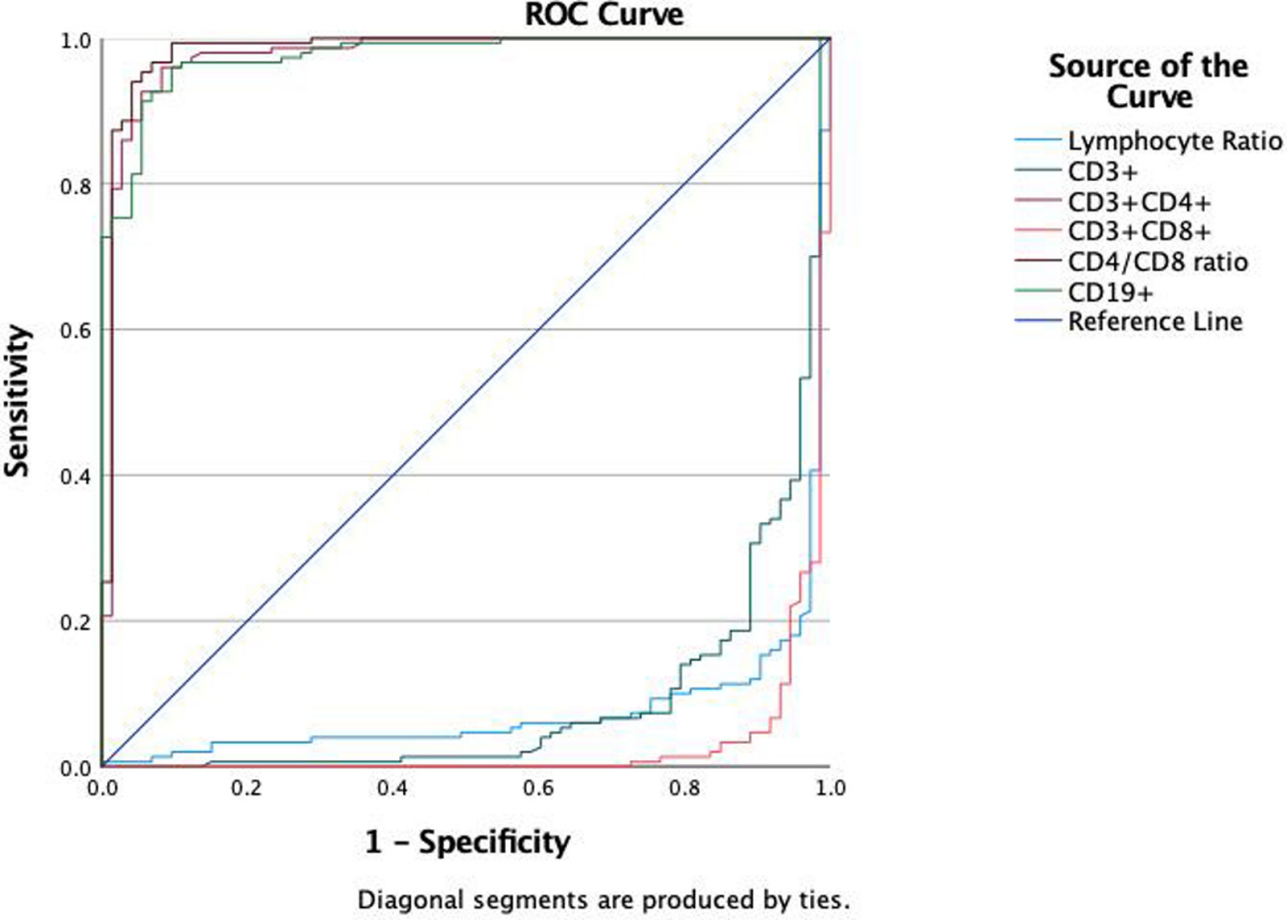
ROC curves of classification efficacies of immune ratios for MPP vs. IM.

**Figure 3:**
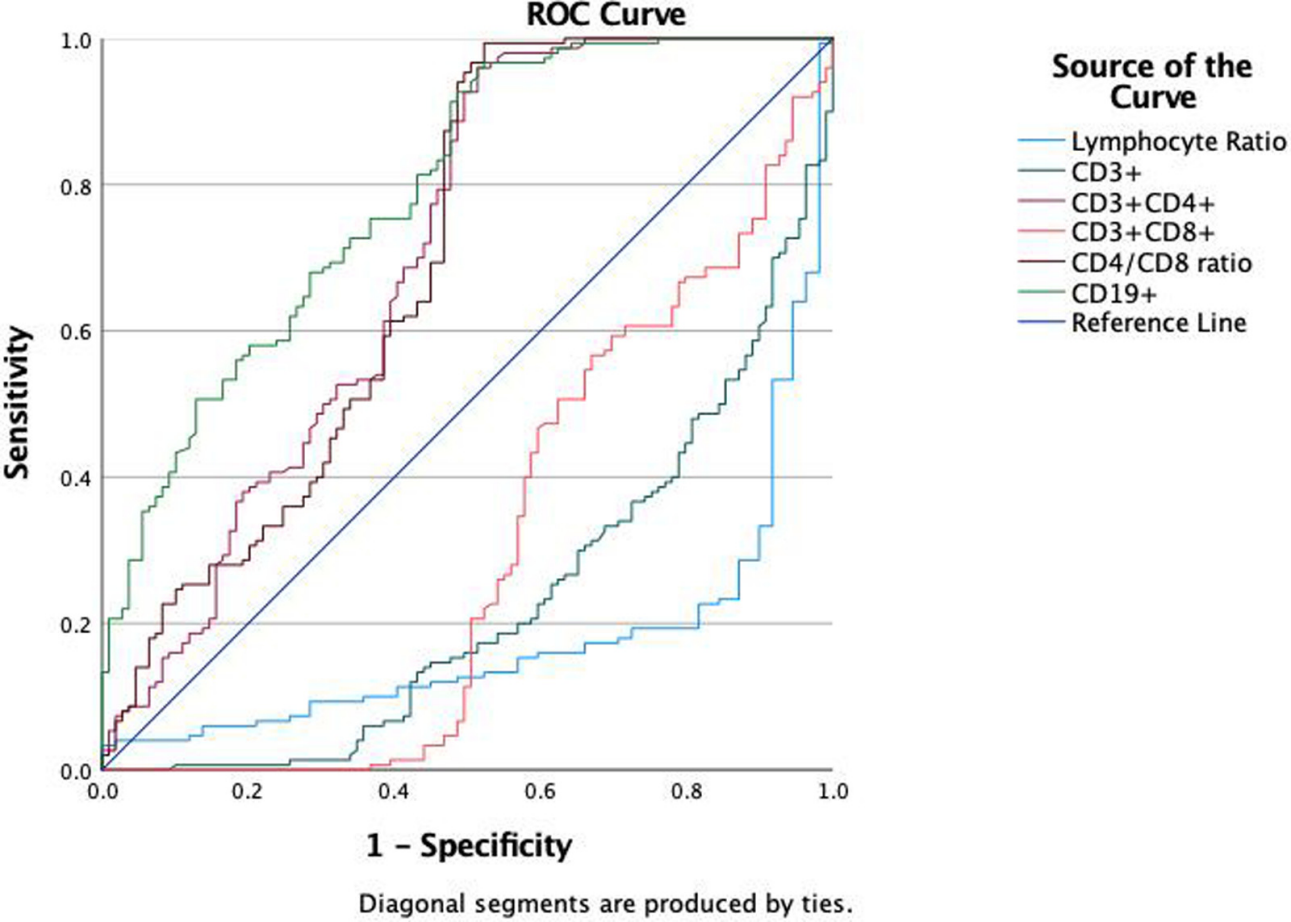
ROC curves of classification efficacies of immune ratios for MPP vs. HC.

We evaluated the diagnostic efficacy of different lymphocyte subsets in distinguishing MPP, IM, and HC through ROC curve analysis (Figures 2, 3). In the comparison between MPP and IM, ROC curve analysis demonstrated that CD3+ T cells (AUC = 0.931), CD3 + CD8+ T cells (AUC = 0.989), CD3 + CD4+ T cells (AUC = 0.992), CD4/CD8 ratio (AUC = 0.996), lymphocyte ratio (AUC = 0.925), and CD19+ B cells (AUC = 0.978) all exhibited excellent diagnostic value (Figure 2, Table 2). Among these, the CD4/CD8 ratio had the highest AUC value (0.996), with a Youden index of 0.951, indicating its excellent diagnostic value (Table 2). The sensitivity and specificity of CD4+ T cells and CD8+ T cells both exceeded 0.9, with Youden indices of 0.921 and 0.899, indicating a high diagnostic efficacy (Table 2). In contrast, CD16 + cells had a relatively low AUC value of 0.541 and a Youden index of 0.158, indicating relatively poor diagnostic performance (Table 2). In the comparison between MPP and HC, the AUC values for CD3+ T cells, CD3 + CD8+ T cells, CD3 + CD4+ T cells and CD19 were 0.772, 0.698, 0.702, and 0.795 (Figure 3, Table 3). CD16+, WBC, CRP, and PCT showed poor diagnostic efficacy, with *P* values all above 0.05, indicating no significant statistical significance (Tables 2, 3).

**Table 2:** Comparison of immune ratio classification efficacy between MPP and IM.

Test result variable(s)	Area	*P*	95% confidence interval		Cut off	Sensitivity	Specificity	Youden
			Lower bound	Upper bound				
Lymphocyte Ratio	0.925	0.023	0.000	0.881	46.87	0.962	0.821	0.783
CD3+	0.931	0.020	0.000	0.891	73.79	0.906	0.821	0.727
CD4+	0.992	0.004	0.000	0.984	23.09	0.940	0.981	0.921
CD8+	0.989	0.006	0.000	0.978	37.73	0.943	0.955	0.899
CD4/CD8 ratio	0.996	0.003	0.000	0.991	0.71	0.970	0.981	0.951
CD19	0.978	0.009	0.000	0.961	8.59	0.970	0.906	0.876
CD16+	0.541	0.043	0.385	0.456	11.52	0.366	0.792	0.158
WBC	0.819	0.033	0.000	0.753	10.79	0.811	0.746	0.558
CRP	0.585	0.045	0.071	0.496	9.14	0.463	0.736	0.199
PCT	0.728	0.036	0.000	0.657	0.07	0.962	0.470	0.432

**Table 3:** Comparison of immune ratio classification efficacy between MPP and HC.

Test result variable(s)	Area	*P*	95% confidence interval		Cut off	Sensitivity	Specificity	Youden
			Lower bound	Upper bound				
Lymphocyte Ratio	0.827	0.028	0.000	0.772	42.40	0.872	0.761	0.633
CD3+	0.772	0.030	0.000	0.714	73.19	0.596	0.799	0.395
CD4+	0.702	0.035	0.000	0.632	21.40	0.970	0.486	0.456
CD8+	0.698	0.035	0.000	0.629	37.53	0.486	0.955	0.441
CD4/CD8 ratio	0.697	0.036	0.000	0.627	0.54	1.000	0.477	0.477
CD19	0.795	0.028	0.000	0.740	9.47	0.963	0.486	0.449
CD16+	0.521	0.037	0.565	0.449	16.23	0.216	0.908	0.125
WBC	0.590	0.037	0.016	0.517	8.47	0.515	0.725	0.240
CRP	0.692	0.036	0.000	0.622	4.77	0.761	0.716	0.477
PCT	0.566	0.041	0.078	0.486	0.04	0.851	0.459	0.309

## Discussion

IM is an acute infectious disease caused by the EBV that mainly affects adolescents (1, 2). It is characterized by symptoms such as fever, pharyngitis, eyelid edema, and enlargement of lymph nodes, the liver, and the spleen. While IM is typically a self-limiting condition with a favorable prognosis, rare complications such as hemolytic anemia, neurological disorders, and malignant tumors may occur. Timely diagnosis and effective therapeutic intervention are essential for IM patients, especially pediatric patients.

In this study, we analyzed lymphocyte subsets and CBC parameters in the enrolled population. Significant differences were found in CD3+, CD4+, CD8+, CD19+ T cells, and lymphocyte counts among the MPP, IM, and HC groups, with distinct patterns between IM and HC. These markers may serve as diagnostic indicators. Further analysis showed that CD4+ T cells were reduced, while CD8+ T cells were elevated in IM patients, leading to a lower CD4+/CD8 + ratio. This T-cell subset imbalance may alter immune responses and disease progression. The findings indicate that abnormalities in T cell subsets could be linked to IM pathogenesis and may serve as diagnostic biomarkers.

MP infection induces immune and inflammatory damage in the host, and the severity of MPP is closely tied to the host's innate immune response to MP (26, 27). Prior research has indicated that the pathogenesis of MP infection involves excessive immune responses (18, 28). Our immune phenotyping analysis demonstrated that, compared with the HC group, the MPP group exhibited significantly reduced CD3 + and CD8 + cell counts, while the CD4 + cell count was significantly elevated, resulting in a significantly higher CD4/CD8 ratio. CD4+ T cells contribute to the immunopathology of MPP, while CD8+ T cells may help modulate inflammatory responses (29). This imbalance in T cell subsets is a notable feature in MPP patients. Moreover, the MPP group had a significantly increased CD19 + cell count and a significantly decreased lymphocyte percentage, further supporting the presence of immune dysregulation. Laboratory tests revealed that, compared with the HC group, the MPP group had significantly higher levels of WBC and PCT, reflecting systemic inflammation.

ROC curve analysis to evaluate the diagnostic ability of different lymphocyte subgroups in distinguishing MPP and IM. The results showed excellent diagnostic value with the AUC values of CD3 + CD4+ (AUC = 0.992), CD3 + CD8+ T cells (AUC = 0.989), CD4/CD8 ratio (AUC = 0.996), lymphocyte ratio (AUC = 0.925) and CD19 (AUC = 0.978) respectively. These findings suggest that specific lymphocyte subgroups and related indicators can serve as potential biological markers for differentiating disease states, offering a valuable reference for clinical diagnosis.

White blood cells serve as sensitive markers of infection and inflammation, and peripheral blood monocyte counts correlate with MPP disease severity (30, 31). Monocyte-related inflammatory mechanisms also play a role in its pathogenesis (32, 33). Our findings revealed that WBC and PCT levels were significantly higher in the IM group than in the MPP group, suggesting that the IM group may experience more pronounced inflammation or a higher risk of infection.

Immune cell analysis showed that MPP and IM share common immune system abnormalities, such as alterations in T cell subsets and changes in the CD4+/CD8+ T cell ratio, which could serve as shared diagnostic markers. The proportion of CD3 + cells was significantly higher in the IM group compared to the healthy control group but slightly lower in the MPP group. The proportion of CD19+ B cells was significantly lower in the IM group and higher in the MPP group compared to the healthy control group. Monitoring disease-specific lymphocyte subsets and related indicators could act as potential biomarkers to distinguish between these two disease states, providing valuable insights for clinical diagnosis.

The standard diagnostic methods for MPP primarily include nucleic acid amplification tests (NAATs), such as PCR, which are highly sensitive and specific for detecting M. pneumoniae DNA from respiratory samples. Serological tests detecting antibodies (IgM and IgG) and culture methods are also used but have limitations, including delayed results and cross-reactivity. In contrast, our study utilized flow cytometry to analyze peripheral blood lymphocyte subsets, which provides a rapid and complementary diagnostic tool. This method does not replace standard tests but serves as an additional approach to enhance diagnostic accuracy by assessing the immune response, which can help differentiate MPP from other similar infections.

The limitation of this study is its single-center design with relatively small sample size. The correlation between the CD4+/CD8 + ratio and the severity of IM and MPP were not analyzed or compared. Furthermore, this study lacked longitudinal follow-up data, preventing assessment of lymphocyte subset dynamics during recovery or correlation with long-term outcomes. Additionally, there was a significant age disparity between the healthy control group (HC) and both the IM and MPP groups. As lymphocyte subset proportions are known to vary with age, this discrepancy may have confounded the comparisons between groups. Future research could adopt more analyzed prospective study designs and involve multiple centers to overcome these limitations.

## Conclusion

Peripheral blood lymphocyte subset analysis serves as a precise and efficient diagnostic tool to directly and unambiguously distinguish between MPP and IM. Integrating peripheral blood lymphocyte testing into diagnostic protocols enables clinicians to improve the accuracy and assurance of their diagnoses, thereby facilitating timely intervention and more effective patient care.

## Data Availability

The original contributions presented in the study are included in the article/Supplementary Material, further inquiries can be directed to the corresponding authors.
